# The incidence of ectopic/heterotopic pregnancies after blastocyst-stage frozen-thawed embryo transfers compared with that after cleavage-stage: a Society for Assisted Reproductive Technologies Clinical Outcomes Reporting System study

**DOI:** 10.1016/j.xfre.2021.06.010

**Published:** 2021-07-03

**Authors:** Kavitha Krishnamoorthy, Patricia Greenberg, Barry E. Perlman, Sara S. Morelli, Sangita K. Jindal, Peter G. McGovern

**Affiliations:** a Obstetrics, Gynecology and Reproductive Health, Rutgers New Jersey Medical School, Newark, New Jersey; b Department of Biostatistics and Epidemiology, Rutgers School of Public Health, Piscataway, New Jersey; c Montefiore’s Institute for Reproductive Medicine and Health, Hartsdale, New York; d University Reproductive Associates, Hasbrouck Heights, New Jersey

**Keywords:** Ectopic pregnancy, heterotopic pregnancy, blastocyst-stage FET, cleavage-stage FET

## Abstract

**Objective:**

To investigate whether there is a difference in the ectopic/heterotopic pregnancy rate of blastocyst-stage frozen-thawed embryo transfers (FETs) compared with that of cleavage-stage FETs.

**Design:**

A retrospective cohort study.

**Setting:**

Not applicable.

**Patient(s):**

Women undergoing autologous FETs at either the blastocyst stage (n = 118,572) or the cleavage stage (n = 117,619), as reported to the Society for Assisted Reproductive Technology from 2004 to 2013.

**Intervention(s):**

None.

**Main Outcome Measure(s):**

Pregnancy outcomes, specifically ectopic pregnancy rates and heterotopic pregnancy rates.

**Result(s):**

Among those who became pregnant, there was a significantly lower incidence of ectopic/heterotopic pregnancies in blastocyst-stage FETs versus that in cleavage-stage FETs (0.8% vs. 1.1%). The differences in ectopic/heterotopic pregnancy rates remained statistically significant after controlling for confounders such as tubal factor infertility and number of embryos transferred.

**Conclusion(s):**

Blastocyst-stage FET was associated with a lower ectopic/heterotopic pregnancy rate compared with cleavage-stage FET.

**Discuss:** You can discuss this article with its authors and other readers at **https://www.fertstertdialog.com/posts/xfre-d-20-00262**

In vitro fertilization (IVF) has expanded the possibilities of conception and resulted in increased successful pregnancies over the past several years (1). With the use of IVF, many pathologies that previously prevented normal pregnancies from occurring have been overcome, including any disruption to the anatomy of the fallopian tubes such as inflammation or obstruction. These tubal pathologies may inhibit normal embryo transport into the intrauterine cavity or result in abnormal implantation into the fallopian tube (2, 3, 4). The incidence of ectopic pregnancy in the general population remains stable, ranging between 0.6% and 2.1% (6.4–20.7 per 1,000 pregnancies) in the United States (5, 6, 7); 90% of ectopic pregnancies occur in the fallopian tube, making this the most common location of pregnancies implanting outside the uterus (8). Risk factors include a history of previous ectopic pregnancy, a history of pelvic surgery, a history of pelvic inflammatory disease, and smoking (9, 10, 11). With the use of IVF, the embryo is transferred directly into the uterine cavity, potentially bypassing any tubal pathology that would otherwise result in failed implantation or ectopic pregnancy. However, the incidence of ectopic pregnancies with the use of IVF remains not insignificant, at up to 8.6% (12, 13, 14); the rate has seemed to decrease in recent years down to 1.6% (15). Some suggested that stimulated cycles lead to increased uterine contractility, which can push a transferred embryo from the uterine cavity into the fallopian tube (16, 17, 18, 19, 20). Others concluded that there may be stronger signals for implantation from the tubal epithelium than from the endometrial epithelium (16, 19).

Although the incidence is much rarer at a rate of 0.03 per 1,000 pregnancies, heterotopic pregnancies share similar risk factors such as smoking, history of ectopic pregnancy, previous pelvic surgery, and inflammation caused by endometriosis and/or pelvic inflammatory disease (21, 22, 23, 24). With the use of assisted reproductive technology (ART), the presence of an intrauterine pregnancy and simultaneous ectopic pregnancy has become more common, 0.26–1.5 per 1,000 ART pregnancies (25, 26, 27). A large contributor to this increased incidence is the transfer of multiple embryos into the intrauterine cavity during IVF, which increases the odds of heterotopic pregnancy by 20-fold, especially when transferring more than 2 embryos at a time (15, 28). Additionally, it is possible that women may spontaneously conceive with intercourse during a natural or modified natural frozen embryo transfer (FET) cycle, which may increase the risk of heterotopic pregnancy (25, 26, 27, 29). This specific risk of heterotopic pregnancy can be mitigated by appropriate patient counseling about abstaining from sexual intercourse during the time of embryo transfer or by preventing natural ovulation with the use of gonadotropin suppression (30, 31).

It is well established that fresh embryo transfers at the blastocyst stage result in improved pregnancy outcomes compared with those of cleavage-stage embryo transfers (32, 33, 34, 35, 36). A blastocyst-stage embryo transfer is defined as the transfer of a day 5–6 embryo, and a cleavage-stage transfer is an embryo transferred on day 2–3 according to the Society for Assisted Reproductive Technologies (SART). Using the SART Clinical Outcomes Reporting System (SART CORS) dataset, we recently showed that blastocyst-stage FET (defined by SART as a transfer occurring after the thawing of a cryopreserved oocyte or embryo) was associated in addition with higher live birth rates compared with those of cleavage-stage FETs (37). However, other outcomes such as ectopic and heterotopic pregnancy rates after cleavage-stage and blastocyst-stage FETs have been inconsistently reported in the literature. Specifically, some studies reported a lower risk of ectopic pregnancy with blastocyst-stage transfers (3, 12, 38, 39, 40, 41). Other studies found no difference in ectopic or heterotopic pregnancy rates between cleavage-stage and blastocyst-stage embryo transfer (13, 15, 25, 42, 43). In contrast, some studies reported that blastocyst-stage transfers might even increase the incidence of ectopic pregnancy (14, 44). Thus, the objective of this study was to investigate, using the SART CORS database, whether there was a difference in the ectopic/heterotopic pregnancy rates for blastocyst-stage FETs compared with those for cleavage-stage FETs.

## Materials and methods

All IVF cycles reported to SART from 2004 to 2013 were evaluated (45). The data were collected and verified by SART and reported to the Centers for Disease Control and Prevention in compliance with the Fertility Clinic Success Rate and Certification Act of 1992 (Public Law 102-493). The data in the SART CORS database are validated annually with some clinics having on-site visits for chart review based on an algorithm for clinic selection. During each visit, the data reported by the clinic were compared with the information recorded in the patients’ charts. Ten out of 11 data fields selected for validation were found to have discrepancy rates of ≤5% (45).

From a total of 256,287 FET cycles from 2004 to 2013 reported to SART, 127,998 cycles resulted in pregnancy. The patients included were those with recorded treatment outcomes and positive pregnancy tests undergoing FETs at either the blastocyst stage (n = 71,855) or the cleavage stage (n = 56,133). All patients who underwent fresh embryo transfers or donor cycles and those with incomplete information reported to SART were excluded. In addition, patients who did not undergo embryo transfer were excluded from the study. Overall, 20,096 cycles met the exclusion criteria and were excluded. The main outcome measures were pregnancy-related outcomes, specifically ectopic pregnancy rates and heterotopic pregnancy rates. The ectopic pregnancy rate was defined as the incidence of a pregnancy in which the embryo(s) implanted outside the uterine cavity per cycle. The heterotopic pregnancy rate was defined as a clinical intrauterine gestation in combination with an ectopic pregnancy per cycle (46). The demographic criteria from each cycle including age at FET cycle start and body mass index (BMI, defined as weight in kilograms divided by height in meters squared) were collected. Other possible clinical confounders such as smoking status, history of tubal ligation, presence of tubal or uterine disease, endometriosis, and number of embryos transferred were analyzed in addition.

Statistical analysis was performed using R: A language and environment for statistical computing (R Foundation for Statistical Computing, Vienna, Austria; https://www.R-project.org) and Microsoft Excel. Pearson’s χ^2^ analyses were used to examine the unadjusted bivariate associations between the FET stage (blastocyst vs. cleavage) and patient demographic and pregnancy characteristics. Multiple logistic regression models with the outcome modeled as ectopic/heterotopic pregnancy (yes/no) and the resulting odds ratios (ORs, with 95% confidence intervals [CIs]) were used to examine the adjusted associations including age, BMI, smoking status, history of tubal ligation, presence of tubal or uterine disease, endometriosis, and number of embryos transferred. All 2-sided *P* values <.05 were considered statistically significant. This retrospective cohort study was approved by the Rutgers Health Sciences Institutional Review Board and the SART Research Committee before data release to our institution.

## Results

A total of 127,998 FET cycles at either the blastocyst stage (n = 71,855) or the cleavage stage (n = 56,133) that resulted in pregnancy were included for analysis. The patient demographic data and pregnancy characteristics for these FET cycles from 2004 through 2013 are shown in Table 1. Any cycle with missing data for age, BMI, smoking status, history of tubal ligation, presence of tubal or uterine disease, endometriosis, and number of embryos transferred was excluded. There were statistically significant differences in age, BMI, smoking status, history of tubal ligation, the presence of tubal hydrosalpinx, endometriosis, uterine disease, as well as the number of embryos transferred between the cleavage-stage and blastocyst-stage groups (*P*<.03); however, there was no clinically significant difference between the groups and these differences can most likely be attributed to the large analytic sample size for both groups.

**Table 1:** Demographics for patients with pregnancies from FET cycles.

	Cleavage-stage group		Blastocyst-stage group	
	Intrauterine pregnancy (n = 55,528)	Ectopic or heterotopic (n = 605)	Intrauterine pregnancy (n = 71,313)	Ectopic or heterotopic (n = 542)
Maternal age at start (y)				
Range	19–44	22–44	18–44	23–44
Mean (SD)	34.3 (4.2)	34.4 (4.3)	34.2 (4.2)	34.3 (4.2)
BMI (kg/m^2^)				
Range	0–50	16.6–45	0–49.9	17.6–44.5
Mean (SD)	25.0 (5.4)	25.7 (5.4)	24.9 (5.4)	25.6 (5.6)
Missing/incorrectly calculated	n = 31,897	n = 335	n = 16,393	n = 89
History of smoking, no. (%)				
No	27,217 (49.0)	308 (50.9)	58,265 (81.7)	460 (84.9)
Yes	2,352 (4.2)	25 (4.1)	3,210 (4.5)	20 (3.7)
Unknown	25,959 (46.8)	272 (45.0)	9,836 (13.8)	62 (11.4)
History of tubal ligation, no. (%)				
No	54,251 (97.7)	597 (98.7)	70,109 (98.3)	539 (99.4)
Yes	1,277 (2.3)	8 (1.3)	1,202 (1.7)	3 (0.6)
Tubal hydrosalpinx, no. (%)				
No	54,700 (98.5)	577 (95.4)	70,389 (98.7)	533 (98.3)
Yes	828 (1.5)	28 (4.6)	922 (1.3)	9 (1.7)
Endometriosis, no. (%)				
No	48,866 (88.0)	533 (89.4)	63,754 (89.4)	488 90.0)
Yes	6,662 (12.0)	72 (10.6)	7,557 (10.6)	54 (10.0)
Uterine disease, no. (%)				
No	52,897 (95.3)	581 (96.0)	67,605 (94.8)	510 (94.1)
Yes	2,631 (4.7)	24 (4.0)	3,706 (5.2)	32 (5.9)
Total no. of embryos transferred				
Range	1–13	1–9	1–15	1–7
Mean (SD)	2.4 (1.0)	2.5 (1.0)	1.8 (0.7)	1.9 (0.8)
Median [Q1,Q3]	2 [2,3]	2 [2,3]	2 [1,2]	2 [1,2]

BMI = body mass index; FET = frozen-thawed embryo transfer.

**Figure 1:**
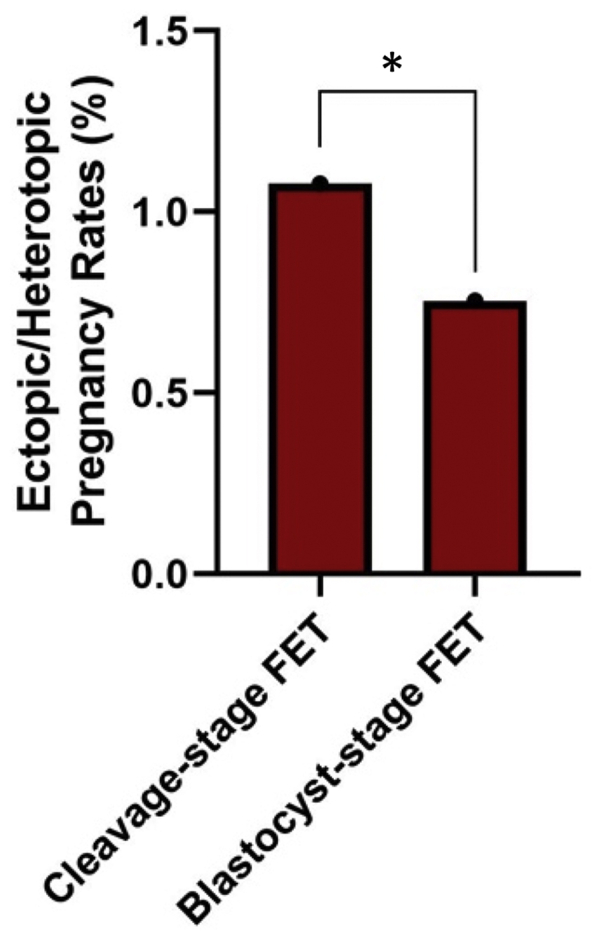
Incidence of cumulative ectopic and heterotopic pregnancy rates in blastocyst-stage vs. cleavage-stage FET cycles that resulteed in pregnancy. Ectopic/heterotopic pregnancy rate (percentage) of cleavage-stage FETs was 1.1% (n = 56,133) versus that of blastocyst-stage FETs, 0.8% (n = 71,855). ∗*P*<.001. FET = frozen-thawed embryo transfer.

Among the FET cycles resulting in pregnancy, there were significantly lower cumulative ectopic and heterotopic pregnancy rates in the blastocyst-stage FETs versus those in the cleavage-stage FETs (0.8% vs. 1.1%; *P*<.001), as shown in Figure 1. In addition, ectopic/heterotopic pregnancy rates remained significantly lower for blastocyst-stage versus cleavage-stage FETs after controlling for other potential confounders through multiple logistic regression analyses (Table 2). After controlling for smoking status, blastocyst-stage transfers resulted in lower ectopic/heterotopic pregnancy rates (OR = 0.69; 95% CI: 0.60–0.80). In addition, there were fewer ectopic/heterotopic pregnancies in blastocyst FETs when controlling for age (OR = 0.70; 95% CI: 0.62–0.78) or BMI (OR = 0.72; 95% CI: 0.62–0.84). Additionally, when controlling for the number of embryos transferred, ectopic/heterotopic pregnancy rates remained significantly lower in blastocyst-stage FETs than those in cleavage-stage transfers (OR = 0.76; 95% CI: 0.67–0.86). Similar findings resulted when controlling for tubal factor infertility including history of tubal ligation (OR = 0.70; 95% CI: 0.62–0.78) or the presence of a hydrosalpinx (OR = 0.70; 95% CI: 0.62–0.79). After controlling for all these factors using multivariate analysis, the rate of ectopic/heterotopic pregnancies remained statistically lower in the blastocyst FETs compared with those in the cleavage-stage FETs (OR = 0.75; 95% CI: 0.63–0.88).

**Table 2:** Adjusted odds ratio for ectopic/heterotopic pregnancy for blastocyst-stage versus cleavage-stage FET.

Confounder used for adjustment	Blastocyst-stage FET vs. cleavage-stage FET Odds ratio (95% CI)
Age (y)	0.70 (0.62–0.78)
BMI (kg/m^2^)	0.72 (0.62–0.84)
Smoking (yes)	0.69 (0.60–0.80)
History of tubal ligation	0.70 (0.62–0.78)
Tubal hydrosalpinx	0.70 (0.62–0.79)
Endometriosis	0.70 (0.62–0.78)
Uterine disease	0.70 (0.62–0.78)
No. of embryos transferred	0.76 (0.67–0.86)
All confounders	0.75 (0.63–0.88)

BMI = body mass index; CI = confidence interval; FET = frozen-thawed embryo transfer.

One patient underwent transfer of 15 cleavage-stage embryos, and another patient underwent transfer of 13 blastocyst-stage embryos. On the basis of the 2017 recommendations from the American Society for Reproductive Medicine (formerly The American Fertility Society) in regards to the number of embryos to be transferred (transfer of no more than 5 embryos at once), the data were recalculated excluding all patients who underwent transfer of more than 5 embryos (30). Differences in ectopic/heterotopic pregnancy rates between blastocyst-stage and cleavage-stage FETs remained statistically significant (Table 3).

**Table 3:** Adjusted odds ratio for ectopic/heterotopic pregnancy for blastocyst-stage versus cleavage-stage FET for patients undergoing transfer of ≤5 total embryos.

Confounder used for adjustment	Blastocyst-stage FET vs. cleavage-stage FET Odds ratio (95% CI)
Age (y)	0.70 (0.62–0.79)
BMI (kg/m^2^)	0.73 (0.62–0.85)
Smoking (yes)	0.69 (0.60–0.80)
History of tubal ligation	0.70 (0.62–0.79)
Tubal hydrosalpinx	0.70 (0.63–0.79)
Endometriosis	0.70 (0.62–0.79)
Uterine disease	0.70 (0.62–0.79)
No. of embryos transferred	0.75 (0.67–0.85)
All confounders	0.75 (0.63–0.88)

BMI = body mass index; CI = confidence interval; FET = frozen-thawed embryo transfer.

## Discussion

The results of this study showed a significantly decreased rate of ectopic or heterotopic pregnancy after blastocyst-stage FET versus that after cleavage-stage FET. A possible explanation for this finding includes better synchronization of the transferred embryo at the blastocyst stage and the receptivity of the endometrium. During a normal spontaneous conception, fertilization and transformation into a cleavage-stage embryo occur in the fallopian tube. The embryo enters the uterine cavity as a morula, where intricate communication between the embryo (now a blastocyst) and the endometrium allows for implantation (47). Transferring an embryo into the uterus at the blastocyst-stage, compared with a cleavage-stage transfer, more closely mimics the stage of the embryo present in the uterus during a naturally spontaneous conception (48). As a result, a blastocyst may correctly implant at a higher rate and location compared with a cleavage-stage embryo transferred at similar time points.

With the extended use of ART over the last half century, the opportunity for pregnancy has expanded greatly. At the same time, abnormal implantations including ectopic and heterotopic pregnancies have increased as well. Both ectopic and heterotopic pregnancies have potentially serious complications, and the continued prevalence of ectopic and heterotopic pregnancies during IVF suggests that various components are involved.

During controlled ovarian stimulation for IVF, sex hormones, estrogen and progesterone, rise to supraphysiologic levels. Both estrogen and progesterone play key roles in regulating embryo movement in the fallopian tube and implantation (49, 50). To avoid the potential adverse effects of very high levels of estrogen and progesterone on the tubal and intrauterine epithelium, a preferential shift toward use of frozen-thawed embryo transfers over fresh embryo transfers has recently emerged (33, 34, 35, 36, 37).

On a similar note, a shift in practice from transferring cleavage-stage embryos to blastocyst-stage embryos has occurred as well. In spontaneous conception, the embryo is usually present in the fallopian tube during the cleavage stage of development and progressively moves into the intrauterine cavity as it transforms into a blastocyst (47). Numerous studies have shown that blastocyst-stage embryo transfer yields better live birth rates, improved cycle outcomes, and decreased miscarriage rates compared with those of cleavage-stage embryo transfer (3, 37, 51). Furthermore, our group recently showed, using the SART CORS database, that blastocyst-stage FET had superior live birth rates compared with those of cleavage-stage FET (37).

The strengths of our study included its large sample size, controlling for multiple possible confounding variables, and the wide range of ages that similarly represent the general population of reproductive women. Our results in this study demonstrated a significantly lower incidence of ectopic/heterotopic pregnancy after blastocyst-stage FET compared with that of cleavage-stage FET. This result remained statistically significant after controlling for the patients’ history of smoking, presence of hydrosalpinx, history of tubal ligation, history of uterine pathology, and the number of embryos transferred. These findings were similar to those previously reported by smaller studies comparing ectopic pregnancy rates in blastocyst FET with those in cleavage-stage fresh transfer (3, 18, 39, 41). However, data on the incidence of heterotopic pregnancy rates after blastocyst-stage embryo transfer versus cleavage-stage FET are sparse; and quite similarly, the overall incidence of heterotopic pregnancy in our study was very low. To our knowledge, this is the first study comparing both ectopic and heterotopic pregnancy rates in a large number of patients undergoing either blastocyst-stage or cleavage-stage FETs. These results should further encourage the shift of practice toward blastocyst-stage FETs.

Our study has limitations that must be addressed. These include its retrospective nature and the limitations of the SART CORS data that are autopopulated by SART member clinics. For example, BMI and smoking were not well-populated fields in this cohort, as demonstrated by the vast number of patients without BMI or smoking history recorded. As a result, these cycles were excluded from the analysis. Furthermore, although the study included a large sample size, most but not all clinics in the United States report to SART. The clinic-specific protocols and processes for embryo transfer along with the protocols used for cryopreservation are unknown. Additionally, the locations of pregnancies and how the diagnosis of either ectopic or heterotopic pregnancy was made are unknown. At the time of this data collection, both ectopic and heterotopic pregnancy rates were reported together. Given the different pathologies and live birth rates between ectopic and heterotopic pregnancies, reporting the ectopic and heterotopic pregnancy outcomes separately would be useful. Additionally, this information would be important for counseling and management strategies of these abnormal pregnancies and should be included in future studies.

As expected, the data for number of embryos transferred were not normally distributed. One patient underwent transfer of 13 cleavage-stage embryos and another patient underwent transfer of 15 blastocyst-stage embryos; both scenarios go against the current American Society for Reproductive Medicine recommendations for the number of embryos to transfer. The number of previous IVF attempts made per patient was unknown, and presumably, poorer prognosis patients would have more embryos transferred. In addition, it is unknown whether the FETs were from planned cycles of freeze-all embryos (first transfer) or if the embryos transferred were from planned cycles of fresh transfer with FET performed later on (second transfer). In the latter case, it may be possible that transfer of the highest quality embryo occurred during a fresh cycle, and that lesser, secondary-quality embryos were then left for cryopreservation. Another limitation was that information about the embryo grade was not available, so it was not feasible to control for this potential scenario with the dataset provided because it was unknown if a prior fresh embryo transfer took place.

Additionally, there were changes in the IVF practices and technologies over the course of this dataset period that cannot be controlled for. For example, a large portion of the embryos may have been cryopreserved via the slow-freeze technique instead of using the more up-to-date method of vitrification for cryopreserving embryos (47, 52). In light of this, our findings are limited in the generalizability of lower ectopic/heterotopic pregnancy rates with blastocyst FETs. That being said, several studies comparing thawed transfer of vitrified blastocyst-stage and cleavage-stage embryos supported similar results: blastocyst FETs had a lower rate of ectopic/heterotopic pregnancy compared with that of cleavage-stage FETs (3, 35, 36, 40). Lastly, our analysis did not include the variable of preimplantation genetic testing, despite its growing popularity.

These findings further support the increasing trend of performing blastocyst-stage FET in clinical practice. The benefits of blastocyst-stage FET compared with cleavage-stage FET include higher clinical pregnancy rates, increase in live birth rates, and decreased odds of miscarriage (37). Our study results supported the transfer of frozen-thawed blastocyst-stage embryos over cleavage-stage embryos. Despite the preference for blastocyst FET, some clinics are still performing cleavage-stage FET. Although cleavage-stage transfer may be appropriate in certain clinical settings (i.e., history of poor blastocyst conversion), providers should consider blastocyst transfer over cleavage-stage transfer, when possible (53). Our results should encourage providers to continue with the inclination of blastocyst FETs over cleavage-stage FETs to decrease the incidence of ectopic/heterotopic pregnancies.

## Conclusion

Blastocyst-stage FET was associated with lower ectopic/heterotopic pregnancy rates compared with those of cleavage-stage FET. These significant findings have the potential to enhance future counseling regarding the optimal timing of embryo transfer.
